# Association of HCV with diabetes mellitus: an Egyptian case-control study

**DOI:** 10.1186/1743-422X-8-367

**Published:** 2011-07-26

**Authors:** Eman I Elhawary, Gamal F Mahmoud, Mai A El-Daly, Fatma A Mekky, Gamal G Esmat, Mohamed Abdel-hamid

**Affiliations:** 1 Microbiology Department, Faculty of Pharmacy, Minia University, Minia, Egypt; 2 Molecular Biology Unit, Viral Hepatitis Research Laboratory, (VHRL). 10 Kasr Elainy Street, Cairo, Egypt; 3 Community, Environmental and Occupational Medicine Department, Faculty of Medicine, Ain Shams University, Cairo, Egypt; 4 Tropical Diseases Department, Faculty of Medicine, Cairo University, Cairo, Egypt

## Abstract

**Background:**

The highest Hepatitis C Virus (HCV) prevalence in the world occurs in Egypt. Several studies from different parts of the world have found that 13% to 33% of patients with chronic HCV have associated diabetes, mostly type II Diabetes Mellitus (DM). In Egypt the prevalence of DM is 25.4% among HCV patients. Therefore, it is important to identify the magnitude of the problem of diabetes in order to optimize the treatment of chronic hepatitis C.

**Methods:**

The objective of this case-control study was to evaluate the prevalence of DM and other extrahepatic (EH) manifestations among patients with different HCV morbidity stages including asymptomatic, chronic hepatic and cirrhotic patients. In this study, 289 HCV patients older than 18 were selected as cases. Also, 289 healthy controls were included. Laboratory investigations including Liver Function tests (LFT) and blood glucose level were done. Also serological assays including cryoglobulin profile, rheumatoid factor, antinuclear antibody, HCV-PCR were performed.

**Results:**

Out of 289 HCV cases, 40 (13.84%) were diabetic. Out of 289 healthy controls, 12 (4.15%) were diabetic. It was found that the diabetic HCV group mean age was [48.1 (± 9.2)]. Males and urbanians represented 72.5% and 85% respectively. Lower level of education was manifested in 52.5% and 87.5% were married. In the nondiabetic HCV group mean age was [40.7 (± 10.4)]. Males and urbanians represented 71.5% and 655% respectively. secondary and higher level of education was attained in 55.4% and 76.7% were married. Comparing between the diabetic HCV group and the non diabetic HCV group, age, residence and alcohol drinking were the only significant factors affecting the incidence of diabetes between the two groups. There was no significant difference regarding sonar findings although cirrhosis was more prevalent among diabetic HCV cases and the fibrosis score was higher in diabetic HCV patients than among the non diabetic HCV cases.

**Conclusion:**

The diabetic patients in the HCV group were older, more likely to have a history of alcohol drinking than the non diabetic HCV cases. Age and alcohol drinking are factors that could potentially contribute to the development of type 2 diabetes. Logistic regression analyses showed that age and residence in urban regions were the predictive variables that could be associated with the presence of diabetes. Alcohol consumption was not a significant predictive factor.

## Background

The highest Hepatitis C Virus (HCV) prevalence in the world occurs in Egypt at an estimated 12% among the general population [1] and reaches 40% in persons 40 years of age and above in rural areas [2]. HCV Genotype 4 is the predominant genotype being isolated from up to 91% of HCV-infected persons in Egypt [3]. The origin of the HCV epidemic in Egypt has been attributed to intravenous schistosomiasis treatment in rural areas in the 1960s-70s [4].

Although HCV targets at the liver, it has become interestingly evident that HCV can induce diseases of many organs. Cacoub *et al*. [5] reported that 38% of patients with HCV would manifest at least one extrahepatic manifestation during the illness.

Extrahepatic (EH) manifestations associated with HCV infection include endocrinological manifestations such as diabetes mellitus (DM) [6] and thyroiditis [7], rheumatologic manifestations such as arthralgias, arthritis [8] and mixed cryoglobulinemia [9]. The prevalence of clinically significant EH manifestations is relatively low, but can be associated with significant morbidity and even mortality. An awareness and recognition of these manifestations is of paramount importance in facilitating early diagnosis and management of these complications [10].

Type 2 diabetes (T2D) is a major public health problem worldwide [11] as people become more obese and live a more sedentary lifestyle [12]. This is in agreement with studies on T2D in noninfected individuals as well as patients infected with other HCV genotypes. The following risk factors are strongly associated with T2D: family history, body fat distribution, age, sex, smoking, and physical activity [13].

Several studies from different parts of the world have found that 13% to 33% of patients with chronic HCV have associated diabetes, mostly type II DM [14]. Diabetes was more frequent in patients having liver cirrhosis than those having chronic hepatitis [15]. Thus DM appears to be a unique EH manifestation of chronic HCV infection [16].

In Egypt the prevalence of DM was 25.4% among HCV patients [17]. Chronic hepatitis C patients are three times more likely to develop DM than HCV seronegative patients [17,14]. Therefore, it is important to identify the magnitude of the problem of diabetes in order to optimize the treatment of chronic hepatitis C.

The objective of this study was to evaluate the prevalence of DM and other EH manifestations among patients with different HCV morbidity stages including asymptomatic, chronic hepatic and cirrhotic patients age and sex matched.

## Patients and methods

### Study design

The current research represents a case control study where patients with chronic HCV infection attending the outpatient clinic of Kasr El-Aini Hospital, Cairo University (KAH), National Hepatology and Tropical Medicine Research Institute (NHTMRI) and Viral Hepatitis Research Laboratory (VHRL) were interviewed. Patients were subjected to a questionnaire to screen those having EH manifestations and general examination. Patients were referred to rheumatologist, dermatologist for further assessment according to their clinical complaints. Laboratory investigations included complete blood picture, liver function tests and blood glucose. Abbott AxSYM System HCV version 3.0 was used for HCV Ab detection followed by In-house RT-PCR [18] for confirmation. Liver function tests (LFT) were carried out using Beckman Synchron CX4 Delta Clinical System (U.S.A.). Serological assay included cryoglobulin profile, rheumatoid factor, antinuclear antibody and HCV-PCR to assess viral load. Abdominal ultrasonography and biopsy was available from some of the patients.

Only HCV patients who were elder than 18 years and had their antibody profile positive for HCV were included as cases. Patients with decompensated liver disease, cancer, on interferon therapy, having end stage renal disease or coexisting viral infection like hepatitis B surface antigen positive patients, pregnant females were excluded from the research. Controls were with normal liver function tests, no serological evidence of HCV and no recent illness.

Defining type 2 diabetes was done according to the American Diabetes Association guidelines (2008)[19]. The current research was approved by the ethics committee of the NHTMRI. All patients gave informed written consents prior to participation and the study was conducted in accordance with Helsinki declaration.

### Statistical Analysis

Comparisions between groups (cases and controls) were done using the Student's *t *test for continuous variables and x^2 ^test was used for nominal categorical variables. Continuous variables were summarized as mean ± SD and categorical variables as frequency and percentage, unless otherwise stated.

All analyses were performed with SPSS software for Windows, version 13 (SPSS Inc., Chicago, IL). Correlation was determined by Pearson's linear regression analysis. All *P *values are based on a two-sided test of statistical significance. P value of ≤ 0.05 will be considered as significant. Logistic regression analyses were used to evaluate the predictive variables that could be associated with the presence of diabetes.

## Results

### I-HCV cases

The mean age of cases (± Standard deviation) was 41.7 (± 10.6) with age range 19 to 65 years old (Table 1). 71.6% were males and 28.4% females. Over two-thirds (68.2%) of cases were from urban regions with 45.7% of them did not complete their secondary education. Regarding their occupation, most of the study sample were skilled workers (29.1%), employees (22.8%), housewives (20.4%), students (1.0%) and others (26.9%).21.4% of them were current smokers, 5.2% were shisha smokers and 7.0% drinked alcohol.

**Table 1:** Sociodemograhic characteristics and special habits of chronic hepatitis C cases.

Characteristic	HCV cases N = 289 No (%)
**Age (years)**	
-Mean ± SD	41.7 ± 10.6
-Range	19-65

**Gender**	
-Male	207 (71.6)
-Female	82 (28.4)

**Residence**	
-Urban	197 (68.2)
-Rural	92 (31.8)

**Level of education**	
-Less than secondary	132 (45.7)
-Secondary & more	157 (54.3)

**Marital status**	
-Not married	63 (21.8)
-Married	226 (78.2)

**Occupation**	
-Skilled workers*	84 (29.1)
-Employees	66 (22.8)
- Student	3 (1.0)
-Housewife	59 (20.4)
-Others	77 (26.6)

**Cigarette Smoking**	60 (21.4)

**Shisha Smoking**	15 (5.2)

**Alcohol Drinking**	5 (7.0)

* skilled workers include farmer, builder, industrial, trade workers.

At least one EH manifestation was shown in 63.3% of HCV cases. Cryoglobulinemia was found positive in 22 patients (7.6%). Rheumatologic manifestations were in 18.4% and dermatologic manifestations in 9.6% of the HCV cases. Xerostomia and pruritis were the most prevalent rheumatologic and dermatological manifestation respectively.

### II- Diabetes mellitus in chronic HCV cases

The mean age of HCV diabetic cases (± SD) [48.1 (± 9.2)] was statistically significantly higher than that of HCV nondiabetics [40.7 (± 10.4)] (P value = 0.001). Gender, level of education, marital status and occupation did not affect the incidence of diabetes among HCV cases (P values = 0.90, 0.35, 0.13 and 0.46 respectively). However, current residence did affect the incidence of DM. 85% of diabetic HCV cases and 65.5% of non diabetic HCV cases were from urban regions and this difference was statistically significant. Regarding special habits, diabetic HCV cases were more often found to smoke shisha & drink alcohol than non diabetics (Table 2). However, they smoke less than nondiabetic HCV cases. The difference was statistically significant regarding alcohol drinking only.

**Table 2:** Sociodemograhic characteristics and special habits of diabetic and nondiabetic HCV cases.

Parameters	HCV Cases		Total N = 289	P-value
	Non diabetic (N = 249) No (%)	Diabetic (N = 40) No (%)		
**Age (years)**				
**-Mean ± SD**	40.7 ± 10.4	48.1 ± 9.2	41.7 ± 10.6	**0.001**
**-Range**	19-65	29-65	19-65	

**Gender**				
**-Male**	178 (71.5)	29 (72.5)	207 (71.6)	0.90
**-Female**	71(28.5)	11(27.5)	82 (28.4)	

**Current residence**				
**-Urban**	163 (65.5)	34 (85.0)	197 (68.2)	**0.01**
**-Rural**	86 (34.5)	6 (15.0)	92 (31.8)	

**Level of education**				
**-Less than secondary**	111 (44.6)	21 (52.5)	132 (45.7)	0.35
**-Secondary & more**	138 (55.4)	19 (47.5)	157 (54.3)	

**Marital status**				
**-Not married**	58 (23.3)	5 (12.5)	63 (21.8)	0.13
**-Married**	191 (76.7)	35 (87.5)	226 (78.2)	

**Occupation**				
**-skilled workers**	77 (30.9)	7 (17.5)	84 (29.1)	
**-employee**	56 (22.5)	10 (25.0)	66 (22.8)	
**- student**	3 (1.2)	0	3 (1.0)	0.46
**-housewife**	46 (18.5)	13 (32.5)	59 (20.4)	
**-others**	67 (26.9)	10(25.0)	77 (26.6)	

**Cigarette Smoking**	53 (22.0)	7 (17.5)	60 (21.4)	0.64

**Shisha Smoking**	11 (4.6)	4 (10.5)	15 (5.2)	0.27

**Alcohol Drinking**	2 (0.8)	3 (7.9)	5 (7.0)	**0.02**

N.B. Skilled workers include (farmer, builder, industrial, trade work).

Diabetic cases were more prone to have cirrhosis than nondiabetic HCV cases. Also their stage of liver fibrosis and inflammation was more severe than nondiabetic HCV cases. However, the difference was not statistically significant between diabetic and nondiabetic HCV cases regarding liver condition. There was no significant difference between diabetic and non diabetic HCV cases regarding the laboratory findings (Table 3).

**Table 3:** Laboratory findings of diabetic and nondiabetic HCV cases.

Laboratory findings	HCV Cases		P-value
	Non diabetic No (%)	Diabetic No (%)	
**Abnormal Liver function**			
**• High ALT**	174 (76)	34 (89.5)	0.06
**• High AST**	170 (75. 2)	24 (64.9)	0.18
**• High ALKP**	21 (19.1)	5 (25)	0.76
**• High BIL**	31 (17.5)	8 (26.7)	0.24

**Abnormal Hemogram**			
**• Anemia**	29 (14.6)	4 (12.1)	0.92
**• Thrombocytopenia**	44 (23.4)	13 (41.9)	0.07
**• Leukopenia**	48 (24.9)	9 (27.3)	0.99
**• Neutropenia**	60 (39.5)	9 (36.0)	0.74
**• Lymphopenia**	9 (5.5)	1(3.2)	0.91

**Autoimmune markers**			
**• Positive ANA**	10 (11.8)	2 (11.1)	0.75
**• Positive RF**	79 (59.0)	13 (52.0)	0.67

**High Alpha-Feto protein**	21(21)	3(3)	1.00

**High Blood GLUC**	0	40 (100)	**0.0001**

**High Glycosylated Hb**	3 (60)	7 (58.33)	0.633

**High TSH**	3 (2.77)	0	0.811

### III. Case control study. HCV cases and non HCV controls

The mean age of non diabetic HCV cases (± Standard deviation) was 40.7 (± 10.4) with age range 19 to 65 years old which was less than the diabetic HCV cases. Their mean age (± Standard deviation) was 48.1 (± 9.2) with age range 29 to 65 years old. The mean age of non diabetic controls (± Standard deviation) was 41.6 (± 11.9) with age range 21 to 65 years old. Diabetic nonHCV controls were elder in age than non diabetic controls (Table 4). Table 4 also shows that age distribution, gender, current residence, level of education, and marital status differed significantly among HCV cases and nonHCV controls regarding diabetic groups.

**Table 4:** Sociodemograhic characteristics of diabetic and nondiabetic HCV cases and diabetic and nondiabetic nonHCV controls.

Parameters	HCV Cases		Controls		P-value
	Non diabetic (N = 249) No (%)*	Diabetic (N = 40) No (%)	Non diabetic (N = 277) No (%)*	Diabetic (N = 12) No (%)	
**Age (years)**					
**-Mean ± SD**	40.7 ± 10.4	48.1 ± 9.2	41.6 ± 11.9	48.6 ± 8.4	**0.001**
**-Range**	19-65	29-65	21-65	34-65	

**Age distribution**					
**-18-30**	52 (20.9)	2 (5.0)	65 (23.5)	0	**0.0001**
**-31-40**	68 (27.3)	7 (17.5)	75 (27.1)	2 (16.7)	
**-41-50**	81 (32.5)	15 (37.5)	70 (25.3)	4 (33.3)	
**51-60**	45 (18.1)	13 (32.5)	49 (17.7)	5 (41.7)	
**-above 60**	3 (1.2)	3 (7.5)	18 (6.5)	1 (8.3)	

**Gender**					
**-Male**	178 (71.5)	29 (72.5)	154 (55.6)	2 (16.7)	**0.0001**
**-Female**	71 (28.5)	11 (27.5)	123 (44.4)	10 (83.3)	

**Current residence**					
**-Urban**	163 (65.5)	34 (85.0)	0	0	**0.0001**
**-Rural**	86 (34.5)	6 (15.0)	277 (100)	12 (100)	

**Level of education**					
**-Less than secondary**	111 (44.6)	21 (52.5)	177 (63.9)	12 (100)	**0.0001**
**-Secondary & more**	138 (55.4)	19 (47.5)	100 (36.1)	0	

**Marital status**					
**-Not married**	58 (23.3)	5 (12.5)	44 (15.9)	5 (41.7)	**0.03**
**-Married**	191 (76.7)	35 (87.5)	233(84. 1)	7 (58.3)	

Regarding blood sugar status, it was found that 53 (7.7%) patients were found to be diabetic while 638 (92.3%) were non diabetic (Table 5). The difference was statistically significant (P = 0.0001). Diabetic cases represented 13.8% of the HCV cases while diabetic controls represented 4.2% of the controls.

**Table 5:** Blood sugar status of case and control study groups.

Blood sugar status	No of patients N° (%)		Total	P-value
	Cases	Controls		
**Diabetic**	40 (13.8)	12 (4.2)	52 (7.7)	**0.0001**
**Non diabetic**	249 (86.2)	277(95.8)	526(92.3)	
**Total**	289 (100)	289(100)	578 (100)	

Abnormal high ALT, high ALKP and high BIL values was more frequently shown among HCV diabetic cases compared with HCV nondiabetic cases (Table 6). In non HCV controls, diabetic subjects less frequently showed high AST and ALT. The difference was statistically significant regarding the former parameters.

**Table 6:** Laboratory findings of HCV cases and controls.

Parameter	Cases		Controls		P-value
	Non diabetic No (%)*	Diabetic No (%)	Non diabetic No (%)*	Diabetic No (%)	
**Abnormal Liver function**					
**• High ALT**	174 (76.0)	34 (89.5)	24 (8.9)	1 (8.3)	**0.001**
**• High AST**	170 (75.2)	24 (64.9)	15 (5.5)	1 (8.3)	**0.001**
**• High ALKP**	21(19.1)	5(25.0)	0	0	**0.02**
**•High BIL**	31 (17.5)	8 (26.7)	0	0	**0.01**

**High Blood GLUC**	0	40 (100)	0	12 (100)	**0.0001**

### IV. HCV Diabetics and non HCV Diabetics

The mean age of diabetic HCV cases (± SD) was 48.1 (± 9.1) with age range 29 to 65 years old. More than one -third of cases fall in the age group 41 to 50 years old and 7.5% only above 60 years old (Table 7). The mean age of diabetic controls (± SD) was 48.6(± 8.4) with age range 34 to 65 years old. Males represented 72.5% of the diabetic HCV cases but only 16.7% of diabetic controls. All of the controls were from rural areas while only 15% of the cases were from rural areas. None of the controls completed their secondary education while 47.5% of the cases did. Table 7 also shows that 87.5% of cases and 58.3% of the controls were married. The difference was statistically significant regarding the former parameters except the marital status.

**Table 7:** Sociodemograhic characteristics of diabetic HCV cases and nonHCV diabetic controls.

Parameters	Diabetic HCV cases (N = 40) N° (%)	Diabetic Controls (N = 12) N° (%)*	P-value
**Age (years)**			
**-Mean ± SD**	48.1 ± 9.1	48.6 ± 8.4	**0.001**
**-Range**	29-65	34-65	

**Age distribution**			
**-18-30**	2 (5.0)	0	**0.0001**
**-31-40**	7 (17.5)	2 (16.7)	
**-41-50**	15 (37.5)	4 (33.3)	
**51-60**	13 (32.5)	5 (41.7)	
**-above 60**	3 (7.5)	1 (8.3)	

**Gender**			
**-Male**	29 (72.5)	2 (16.7)	**0.0001**
**-Female**	11 (27.5)	10 (83.3)	

**Current residence**			
**-Urban**	34 (85.0)	0	**0.0001**
**-Rural**	6 (15.0)	12 (100)	

**Level of education**			
**-Less than secondary**	21 (52.5)	12 (100)	**0.008**
**-Secondary & more**	19 (47.5)	0	

**Marital status**			
**-Not married**	5 (12.5)	5 (41.7)	0.07
**-Married**	35 (87.5)	7 (58.3)	

Regarding abnormal lab characteristics, Table 8 shows that HCV cases were more likely to have high ALT (89.5%) and AST (64.9%) than non HCV controls (8.3% each).

**Table 8:** Abnormal Lab characteristics of diabetic cases and diabetic controls.

Parameters	Diabetic HCV cases N = 40 N° (%)	Diabetic Controls N = 12 N° (%)*	P-value
**High ALT**	34 (89.5)	1 (8.3)	**0.001**

**High AST**	24 (64.9)	1 (8.3)	**0.001**

**High Blood GLUC**	40 (100)	12 (100)	**0.0001**

## Discussion

Several strands of evidence have suggested a possible link between HCV infection and an increased prevalence of Type2D [20]. Based on case-control studies, the prevalence of DM had been reported in 21% to 50% (a two- to ten-fold increase in prevalence) of patients with chronic HCV infection, which was significantly higher than that in the general population or among patients with other forms of liver diseases [21]. This study showed that 13.84% of the HCV patients were type 2 diabetics. These results are comparable to the results of the findings reported by Petit et al. [22], Wang et al. [23] and Veldt et al. [24]. Other authors reported higher numbers of diabetics in their studies on HCV patients as El-Zayadi et al. [17] & Lecube et al. [25]. They reported prevalence of DM to be 20.9%-29% among HCV patients. Prevalences ranging between 32.5% and 39.8% were reported by Zhao et al. [26] and Chehadeh et al. [14]. Differences in the criteria employed in the diagnosis of DM, source of controls, case definition, sample size and underlying target population may explain much of this observed variability among studies.

Diabetes was shown to be prevalent in 4.15% of the controls who represent age-matched normal population. This result (among controls) is 3-4 times less than what was found in this study (among HCV cases), indicating that HCV patients are a high risk population for DM. In contrast, some studies provided evidence against potential association between these two disorders [27]. The prevalence of diabetes in adults in Egypt ranged from 5% in rural communities in the Nile delta to 10% in lower socioeconomic areas of Cairo and over 20% in higher socioeconomic areas in Cairo [28]. Similar results were reported from Italy and India [29,30]. Higher prevalences of DM were reported from other authors [6,26]. This may be attributed to differences in environmental influences, genetic susceptibility and diets.

### Sociodemographic characters and special habits of cases and controls

It was noticed that the highest percentage of diabetes (37.5%) was among age group from 41-50 years which was similar to that reported by Wang et al. [23]. Conversely, others stated that older age was a potential risk factor for development of DM in HCV patients [31]. It is interesting to note that older age is associated with more severe liver disease among HCV-infected patients [32]. Studies from Italy and USA referred that HCV increases the prevalence of DM independently of age [33,34]. These findings have some important clinical and public health implications. They imply that the younger the persons with HCV infection, the greater the risk that they will develop diabetes than will their age-group counterparts without HCV infection. Therefore, screening for and prevention of diabetes in persons with HCV infection could be started earlier than the suggested age of ≥ 45 years for the general population [19], especially for those with higher body mass index levels or with other risk factors for diabetes. In addition, young adults with diabetes in communities with a high prevalence of HCV infection could be tested for an underlying HCV infection.

The mean age for HCV DM+ was higher than the mean age for HCV DM- subjects which is interestingly similar to the findings of Giordanino and coworkers [35]. These findings support the idea that the induction of diabetes in HCV patients is progressive rather than abrupt [14]. Other studies suggest that HCV interferes with glucose metabolism independently of age [21,24].

Diabetes was more prevalent in females than in males. Our findings are similar to that reported by Huang et al. [31]. Males may be more frequently exposed to HCV infection because they have more risk factors including schistosomal infection, shaving beards, accidents, and exposure to operations and blood transfusions [36]. Otherwise, women might be more likely to be associated with HCV clearance and lower rates of HCV-RNA positivity [37]. However, there was no significant difference between diabetic and non-diabetic cases regarding gender which was similar to other studies [24,33]. Persons with type 2 DM tended to have lower educational attainment [38]. This is in concordance with our study.

Habitual smoking is a well-documented risk factor for exacerbation of liver conditions, such as increased alanine transferase levels and increased fibrosis that would increase the likelihood of type 2 diabetes [39]. There was a history of cigarette smoking among 42.2% of HCV subjects which is similar to that reported by Eissa et al. [36]. Our study revealed that HCV cases who were current cigarette smokers were more likely to be diabetic (17.5%) than current shisha or goza smokers (10.5%). Cases who had a history of cigarette smoking (50%) were more likely to be diabetic than those with history of shisha smoking (16.4%). That was in concordance with findings of Alavian et al. [40]. Yet smoking was not a significant predicting factor for diabetes which was similar to results of Wang et al. [23].

Hepatitis C and excessive alcohol consumption are not likely to have an additive effect in relation to the risk of having diabetes [23]. Only 24.6% of the HCV subjects in this study had a history of drinking alcohol and 7.9% were current alcoholics. Current alcohol drinking was a significant risk factor for developing DM which was in agreement with and Ryu et al. [41] findings.

#### Liver condition

Patients with HCV-related liver cirrhosis are associated with a significantly higher prevalence of DM than those cirrhotic patients with other etiologies [42]. This is on line with our study. This study showed that 23.1% of HCV cirrhotics were diabetic which was similar to the findings of other authors [35]. Higher figures were reported by Kwon et al. [43]. The discrepancies among studies may be explained by severity of liver disease. In another remarkable study, in which cirrhotic HCV patients were carefully excluded, a third of them had diabetes [44]. On excluding cirrhotic patients from the HCV cases in this study, 12.5% of the cases had DM which was 4 times the prevalence in the controls representing the normal Egyptian population. Of interest, that was consistent with a study on a large cohort of patients conducted by Lecube and coworkers [25]. Taken together these findings suggest that HCV infection is a more important predictor of glucose intolerance than cirrhosis, and the combination of both factors further increases the risk of diabetes. Other factors than cirrhosis must be found to explain the increased prevalence of DM in HCV patients [22].

Regarding other sonographic features, anti-HCV-positive subjects with sonographic evidence of bright liver, enlarged liver and chronic liver disease had a higher prevalence of T2D compared with patients whose sonographic features were normal. The trend of increasing prevalence of T2D with severity of sonographic stages in anti-HCV-positive subjects implies that viral inflammatory activity, time duration, insulin secretion, insulin sensitivity, and the interaction with other well-known diabetes risk factors appear to play an important role in the development of T2D.

Diabetes is associated with increased fibrosis in chronic HCV but such an association may be related to the high prevalence of diabetes in patients with cirrhosis [45]. The frequency of diabetes mellitus increased along with pathological staging [24]. The fibrosis score was higher in diabetic HCV patients [15]. The results hereafter were on line with that. In this study, none of the diabetic patients had normal stage of fibrosis. Nearly one half of the diabetic patients (46.7%) showed moderate to severe fibrosis compared to 34.7% in non diabetic HCV patients. The discrepancies among studies may be explained by differences in ethnic background, HCV genotype frequency, and duration and severity of liver disease.

Liver biopsy specimens of diabetic HCV patients showed higher inflammatory activity defined by histological activity index score than the nondiabetic HCV group for moderate and severe stages. That is in line with the results reported by several authors [46]. HCV patients who develop diabetes had more severe liver disease according to both their liver enzymes and biopsy findings [22]. These observations suggest that not HCV infection itself but the resultant ongoing inflammation of the liver might determine a higher risk for DM. Patients with an earlier stage of chronic HCV infection have β-cell dysfunction but diabetes does not become established until cirrhosis has supervened [47]. This could explain the negative association between stage of inflammation (found to be normal) and DM manifest in this series.

Blood picture did not differ between the 2 HCV groups. This study was in keeping with the findings of other authors [44]. There was at least one abnormal feature in the hemogram of 68.6% of the HCV cases.

Individuals with type 2 diabetes have a higher incidence of liver function test (LFT) abnormalities than individuals who do not have diabetes [48] which is similar to our findings. However, Chehadeh et al. [14] found no difference in this aspect. Interestingly, diabetic controls in this study had higher mean values of ALT than nondiabetic controls. Studies on European diabetics showed the same results in Greece [49] and Spain [25].

## Conclusion

Despite the close relationship between HCV infection and DM, The underlying mechanism(s) that links diabetes and HCV infection remains conjectural. The increased prevalence of diabetes in chronic hepatitis is unique to HCV and therefore that unique mechanisms may underlie glucose intolerance in HCV patients. Type 2 DM is a complex, multisystem disease with a pathophysiology that includes a defect in insulin secretion, increased hepatic glucose production, and resistance to the action of insulin, all of which contribute to the development of overt hyperglycemia. In addition, obesity, aging, and genetic factors such as family history of DM all may contribute to the development of type 2 DM. All these factors make it difficult to evaluate the pathogenic role of HCV infection in the development of type 2 DM.

Our findings that a more severe inflammatory and fibrotic process were associated with diabetes suggest that the pathogenesis of DM in HCV infection may be multifactorial- a precirrhotic state leading to an abnormal glucose metabolism and insulin resistance, acting in conjunction with undefined pancreatic damage, occurring in genetically prone patients is an explanation. Another possible explanation, as proposed by Caronia et al [47], is that β-cell responsiveness is impaired in patients with HCV, possibly because of direct viral effects on β-cell function. Thus for a given degree of liver dysfunction-and presumably IR, diabetes would be more likely to occur in patients with HCV.

In our study, the diabetic patients in the HCV group were older, more likely to have a history of alcohol drinking than the non diabetic HCV cases, factors that could potentially contribute to the development of type 2 diabetes. Logistic regression analyses showed that age and residence in urban regions were the predictive variables that could be associated with the presence of diabetes. Alcohol consumption was not a significant factor.

## Abbreviations

HCV: Hepatitis C Virus; DM: diabetes mellitus; T2DM: Type 2 diabetes; EH: Extrahepatic; ALT: Alanine transaminase; ALKP: Alkaline phosphatase; BIL: Bilirubin; AST: Aspartate transferase; ADA: American Diabetes Association.

## Competing interests

The authors declare that they have no competing interests.

## Authors' contributions

EIE: Acquisition, analysis and interpretation of data, performed statistical analysis, and practical laboratory work.

GFM: Revising the manuscript for intellectual content.

MED: Carried out practical laboratory work.

FAM: Designer and Primary investigator of the study, performed statistical analysis, and revising the manuscript for intellectual content.

GE: Participated in design and coordination and carried out all clinical aspects of the study.

MAH: Participated in design and coordination and revising the manuscript for intellectual content

All authors read and approved the final manuscript.
